# Learning from the past: Impact of the Arctic Oscillation on sea ice and marine productivity off northwest Greenland over the last 9,000 years

**DOI:** 10.1111/gcb.15334

**Published:** 2020-10-13

**Authors:** Audrey Limoges, Kaarina Weckström, Sofia Ribeiro, Eleanor Georgiadis, Katrine E. Hansen, Philippe Martinez, Marit‐Solveig Seidenkrantz, Jacques Giraudeau, Xavier Crosta, Guillaume Massé

**Affiliations:** ^1^ Department of Earth Sciences University of New Brunswick Fredericton NB Canada; ^2^ Ecosystems and Environment Research Programme (ECRU), and Helsinki Institute of Sustainability Science Helsinki University Helsinki Finland; ^3^ Department of Glaciology and Climate Geological Survey of Denmark and Greenland Copenhagen Denmark; ^4^ Université de Bordeaux CNRS EPHE UMR 5805 EPOC Pessac France; ^5^ Department of Geosciences Aarhus University Aarhus Denmark; ^6^ Université Laval CNRS UMI 3376 TAKUVIK Québec QC Canada; ^7^ Station Marine de Concarneau UMR7159 LOCEAN Concarneau France

**Keywords:** Arctic Oscillation, Baffin Bay, climate change, diatoms, highly branched isoprenoid (HBI) biomarkers, marine sediment, paleoceanography, phytoplankton

## Abstract

Climate warming is rapidly reshaping the Arctic cryosphere and ocean conditions, with consequences for sea ice and pelagic productivity patterns affecting the entire marine food web. To predict how ongoing changes will impact Arctic marine ecosystems, concerted effort from various disciplines is required. Here, we contribute multi‐decadal reconstructions of changes in diatom production and sea‐ice conditions in relation to Holocene climate and ocean conditions off northwest Greenland. Our multiproxy study includes diatoms, sea‐ice biomarkers (IP_25_ and HBI III) and geochemical tracers (TOC [total organic carbon], TOC:TN [total nitrogen], δ^13^C, δ^15^N) from a sediment core record spanning the last c. 9,000 years. Our results suggest that the balance between the outflow of polar water from the Arctic, and input of Atlantic water from the Irminger Current into the West Greenland Current is a key factor in controlling sea‐ice conditions, and both diatom phenology and production in northeastern Baffin Bay. Our proxy record notably shows that changes in sea‐surface conditions initially forced by Neoglacial cooling were dynamically amplified by the shift in the dominant phase of the Arctic Oscillation (AO) mode that occurred at c. 3,000 yr BP, and caused drastic changes in community composition and a decline in diatom production at the study site. In the future, with projected dominant‐positive AO conditions favored by Arctic warming, increased water column stratification may counteract the positive effect of a longer open‐water growth season and negatively impact diatom production.

## Graphical abstract

We present the first continuous and high‐resolution diatom record from northeastern Baffin Bay spanning the last c. 9,000 years. Our data show that the variability in sea ice and primary production on the northwest Greenland shelf is linked to modulations in millennial‐scale Arctic Oscillation. The phenology and diatom community structure is largely controlled by water‐column stratification and sea‐ice conditions.

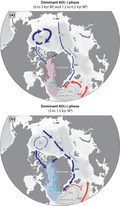

## 1 INTRODUCTION

Climate change is causing an accelerating decline in the seasonal duration and thickness of Arctic sea ice (Serreze & Stroeve, 2015), with important implications for marine primary production (e.g., Bergeron & Tremblay, 2014; Comeau, Li, Tremblay, Carmack, & Lovejoy, 2011; Tremblay et al., 2012). For the Arctic Ocean, satellite‐based measurements suggest that annual net primary production has increased by 30% between 1998 and 2012 (Arrigo & van Dijken, 2015). This increase is largely attributed to thinning sea ice and more abundant and larger melt ponds that allow greater light transmittance and earlier onset of seasonal sea‐ice melt, thereby enhancing both the under‐ice productivity and the length of the growing season (Arrigo et al., 2012; Mundy et al., 2009). However, both satellite observations and in situ measurements indicate pronounced spatial and interannual heterogeneity in the response of primary producers to ongoing Arctic warming owing to local and remote factors that can have a synergistic impact on nutrient supplies and sea‐surface conditions (e.g., ocean currents, vertical mixing, upstream biological processes; Hopwood et al., 2018; Tremblay et al., 2015). Determining future changes in Arctic primary production in a scenario of continued climate warming is therefore challenging, especially since our understanding of how primary production responded to climate forcing on longer timescales is limited by the paucity of comprehensive and highly resolved paleoarchives.

**FIGURE 1:**
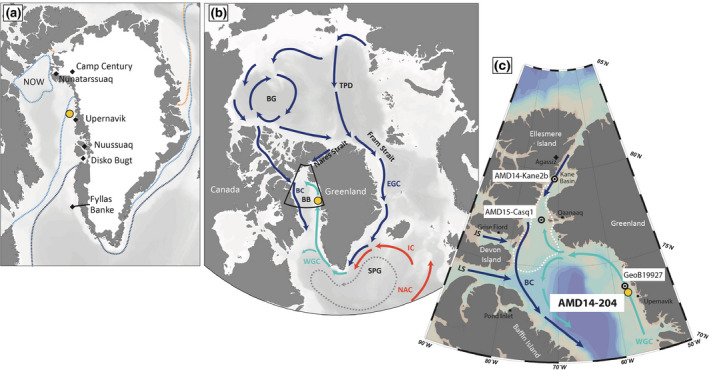
(a) Greenland Ice sheet and historical median sea‐ice extent (1981–2010) for the months of February (dashed dark‐blue line), July (dashed light‐blue line) and September (dashed orange line). Also shown is the July average extent of the North Water polynya (NOW) for the same period. (b) Simplified representation of the main surface‐ocean currents discussed here: The West Greenland Current (WGC) forms by the convergence of the East Greenland Current (EGC) and Irminger Current (IC), a branch of the North Atlantic Current (NAC), as they round the southern tip of Greenland. (c) Location of core AMD14‐204 (yellow circle) studied here, and other cores from northern Baffin Bay and Kane Basin discussed in the text. BB, Baffin Bay; BG, Beaufort Gyre; JS, Jones Sound; LS, Lancaster Sound; NOW, North Water polynya; SPG, Subpolar Gyre; TPD, Transpolar drift

In addition to radiative forcing, variability in hemispheric‐scale atmospheric circulation patterns can impact ecosystem functioning (Post & Forchhammer, 2002). The Arctic Oscillation (AO), also referred to as the Northern Annular Mode, is described as the difference in sea‐level pressure between the Arctic Ocean and the middle latitudes (Cohen & Barlow, 2005; Rigor, Wallace, & Colony, 2002; Thompson & Wallace, 1998). The region defined for AO overlaps with the closely related North Atlantic Oscillation, which measures the regional variability in the atmospheric high‐ and low‐pressure systems over the Azores and Iceland, respectively (Wallace, 2000). Instrumental time series capture a dynamic relationship between the AO and interannual variations in storminess, sea‐surface temperatures and sea‐ice motion and conditions (e.g., Ogi & Wallace, 2007; Rodwell, Rowell, & Folland, 1999; Thompson & Wallace, 2001; Weckström et al., 2013; Zweng & Münchow, 2006). During a positive phase, lower‐than‐normal pressure over the Arctic traps cold air masses in the Arctic, but wintertime surface winds and thermodynamic processes can lead to an overall thinning of the sea‐ice cover over the Arctic Ocean. Therefore, although positive winter AO is associated with a cold anomaly in the surface atmospheric temperature over the Arctic Ocean and Greenland, it counterintuitively results in an earlier Arctic Ocean ice melt and increased export of meltwater and drift ice via the Transpolar Drift through Fram Strait (Rigor et al., 2002) and other gateways such as Nares Strait (Figure 1b). In the northern North Atlantic sector, a positive phase correlates with cold west Greenlandic winters caused by a strengthening of the Baffin Bay and North Atlantic westerlies (Box, 2002). Inversely, negative AO facilitates the buildup of multiyear ice in a strong Beaufort Gyre (Rigor et al., 2002) is associated with reduced export of Arctic freshwater and sea ice via the Arctic gateways, and the prevalence of milder atmospheric winter temperatures along the west Greenland margin. AO can swing between its high and low index polarity within weeks, but persistent modes can also be sustained on decadal to millennial timescales (e.g., Darby, Ortiz, Grosch, & Lund, 2012; Funder et al., 2011; Olsen, Anderson, & Knudsen, 2012). On the Holocene timescale, studies suggest linkages between hemispheric atmospheric circulation and hydrographical conditions in the North Atlantic region (e.g., Andersen, Koç, Jennings, & Andrews, 2004; Giraudeau et al., 2010; Moros, Jansen, Oppo, Giraudeau, & Kuijpers, 2012; Solignac, de Vernal, & Hillaire‐Marcel, 2004; Staines‐Urías, Kuijpers, & Korte, 2013; Van Nieuwenhove, Pearce, Knudsen, Røy, & Seidenkrantz, 2018), and along the Southeast and Western Greenland shelves (e.g., Jennings, Andrews, & Wilson, 2011; Krawczyk et al., 2013; Sha, Jiang, & Knudsen, 2011).

In northern Baffin Bay, oceanographic conditions are influenced by incursions of cold, silica‐ and phosphate‐rich water originating partly from the Pacific and entering Baffin Bay via Nares Strait, Lancaster Sound and Jones Sound (Figure 1), and the northward‐flowing West Greenland Current (WGC; 200–700 m; Straneo, 2006), which is overlain by buoyant meltwater and iceberg supplies from the Greenland Ice Sheet. The WGC is composed of a mixture of cold and low‐salinity polar water carried by the East Greenland Current (EGC), an extension of the Transpolar Drift, and temperate, salty, and nitrate‐rich water derived from the Irminger Current (>3.5°C, >34.88 psu; Myers, Kulan, & Ribergaard, 2007), a branch of the North Atlantic Current (Figure 1b). During intervals of increased export of freshwater and drift ice through Fram Strait, such as during contemporary high index polarity of the AO, a stronger EGC can contribute to a longitudinal expansion of the North Atlantic subpolar gyre, and reduce the northwestward flow of the warm Irminger Water into the WGC (e.g., Flatau, Tally, & Niller, 2003; Morley, Rosenthal, & deMenocal, 2014; Sarafanov, 2009). By contrast, a weaker EGC can be associated with a contracted subpolar gyre and increased northwestward flow of warm Irminger Water into the WGC (e.g., Holland, Thomas, De Young, Ribergaard, & Lyberth, 2008; Jennings et al., 2011). Thus, the dominant mode of atmospheric circulation can indirectly influence the strength and heat advection by the WGC, and amplify changes in surface‐water properties and ocean–ice sheet interactions along the West Greenland margin. At the same time, the AO polarity index influences the consolidation or collapse of the ice arches in Nares Strait (e.g., Georgiadis et al., 2020), thereby also modulating the outflow of Arctic freshwater and drift ice through this gateway.

Given the key role of northern Baffin Bay as mediator between the Arctic and North Atlantic oceans, a number of studies documenting changes in regional ocean conditions in relation to Holocene climate fluctuations have been conducted (e.g., Caron, Rochon, Montero‐Serrano, & St‐Onge, 2019; Caron et al., 2018; Giraudeau et al., 2020; Hansen, Massé, Giraudeau, Pearce, & Seidenkrantz, 2020; Knudsen, Stabell, Seidenkrantz, Eiríksson, & Blake, 2008; Levac, de Vernal, & Blake, 2001; Mudie, Rochon, & Levac, 2005; Saini et al., 2020; St‐Onge & St‐Onge, 2014). However, the link between atmosphere–ocean forcing as expressed by swings in the dominant mode of the AO and changes in sea‐ice seasonality and primary production in northern Baffin Bay has never been investigated. Here, we examine long‐term changes in sea ice and primary production off northwest Greenland, northeastern Baffin Bay, using a series of biogenic proxies applied to a 7.34 m‐long sediment core spanning the last c. 9,000 years. We also provide a continuous and high‐resolution record of changes in diatom production and sea‐ice conditions which can serve as a reference baseline to interpret recent changes and better predict the response of primary producers to future climate change.

## 2 MATERIALS AND METHODS

The marine sediment core AMD14‐204 was collected using a Calypso Square gravity corer, in the cross‐shelf trough north of the Upernavik Ice Stream (73°15.66′N–57°53.98′W, 987 m water depth; Figure 1c), during the 2014 ArcticNet Leg 1b onboard the CCGS *Amundsen*. The core was subsampled onboard using u‐channels and stored at 4°C during transportation. Subsamples for biomarker analyses were stored at −80°C until analysis. Detailed core lithology information is available in Caron et al. (2018).

### 2.1 Age model

**FIGURE 2:**
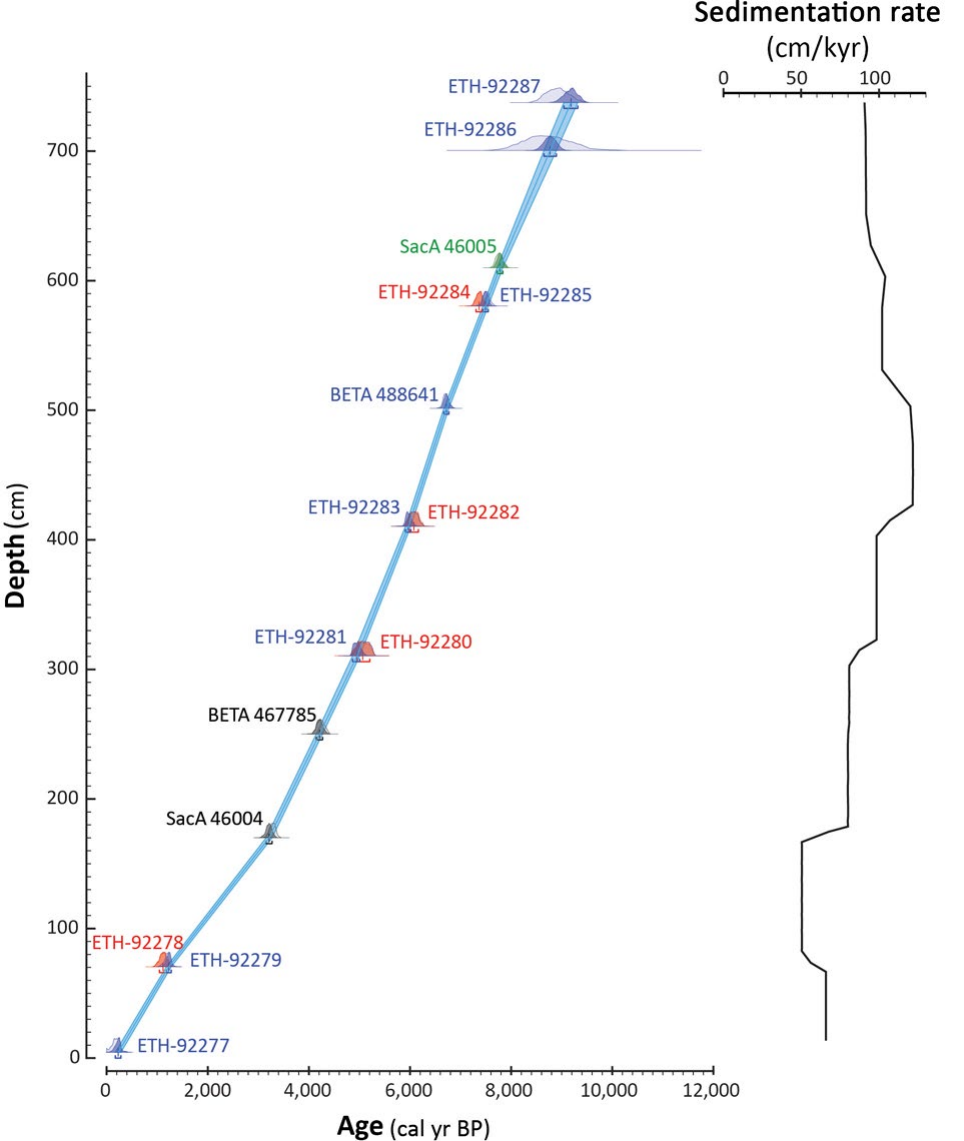
(a) Age‐depth model for core AMD14‐204. The light‐blue envelope represents the modeled 1 sigma range, and the blue line marks the modeled median age. The light‐shaded areas for each radiocarbon date indicate the probability distribution prior to modeling, whereas the darker areas indicate the posterior probability distribution. The color code for the dates indicates the material used: Blue: mixed benthic foraminifera, red: mixed planktonic foraminifera, gray: mixed planktonic and benthic foraminifera, green: mixed ostracods, planktonic, and benthic foraminifera. (b) Sedimentation rate (cm/kyr)

The core chronology is based on 11 accelerator mass spectrometry ^14^C dates from 15 samples of planktic and mixed benthic foraminiferal assemblages, a few also including ostracods (Table 1; Figure 2; Hansen et al., 2020). All dates were calibrated using the Marine13 radiocarbon calibration curve (Reimer et al., 2013) and an additional reservoir age correction (∆R) of 140 ± 30 years was applied (Lloyd et al., 2011). For the age‐depth modeling, a depositional P_sequence model was used with a *k* value of 0.68 (Ramsey, 2008). Based on this age model, the core spans the last 9.2 cal kyr BP (hereafter simply expressed as kyr BP).

**TABLE 1:** Radiocarbon dates for core AMD14‐204

Sample depth midpoint (cm)	Lab. ID	Material	^14^C age (yr BP)	Calibrated age range (cal. yr BP), 1s	Modeled median age (cal. yr BP)	δ^3^C
4.5	ETH‐92277	Mixed benthic foraminifera	705 ± 50	167–276	213	4.94
70.5	ETH‐92279	Mixed benthic foraminifera	1,795 ± 50	1,175–1,270	1,216	4.22
70.5	ETH‐92278	Mixed planktonic foraminifera	1,710 ± 50	1,032–1,175	1,101	6.12
170	SacA 46004	Mixed benthic and planktonic foraminifera	3,555 ± 35	3,139–3,260	3,192	5,7
250.5	BETA 467785	Mixed benthic and planktonic foraminifera	4,300 ± 30	4,133–4,254	4,199	0.3
310.5	ETH‐92281	Mixed benthic foraminifera	4,950 ± 60	4,860–4,992	4,941	3.06
310.5	ETH‐92280	Mixed planktonic foraminifera	4,940 ± 70	4,930–5,188	5,043	5.7
410.5	ETH‐92283	Mixed benthic foraminifera	5,805 ± 60	5,905–6,005	5,959	1.68
410.5	ETH‐92282	Mixed planktonic foraminifera	5,825 ± 60	5,984–6,155	6,063	3.41
501.5	BETA 488641	Mixed benthic foraminifera	6,400 ± 30	6,656–6,751	6,707	−1.8
580.5	ETH‐92285	Mixed benthic foraminifera	7,155 ± 70	7,430–7,531	7,483	2.95
580.5	ETH‐92284	Mixed planktonic foraminifera	7,005 ± 60	7,298–7,417	7,356	2.38
610	SacA 46005	Mixed benthic foraminifera and ostracods	7,445 ± 50	7,712–7,822	7,766	−6,9
700.5	ETH‐92286	Mixed benthic foraminifera	8,270 ± 389	8,639–8,885	8,755	—
737.5	ETH‐92287	Mixed benthic foraminifera	8,489 ± 154	9,017–9,302	9,162	—

### 2.2 Sedimentary proxies

#### 2.2.1 Total organic carbon and nitrogen isotopic analysis

Sedimentary organic carbon is derived from biomass decomposition and can include organic carbon of terrestrial and marine origins. For the total organic carbon (TOC) analyses, the inorganic carbon fraction was removed by adding hydrochloric acid (HCl, 10%) to freeze‐dried samples. HCl was evaporated by heating the samples for 12 hr, TOC was then measured with a LECO C‐S 125 analyzer.

Assuming that diagenetic enrichment (Robinson et al., 2012) of ^15^N is constant through time, the nitrogen isotopic (δ^15^N) signal in marine sediments reflects nitrate (NO_3_^–^) supply and use in the euphotic zone. Light nitrate (^14^NO_3_^–^) is preferentially used by phytoplankton, resulting in higher δ^15^N values when phytoplankton production is elevated. Alternatively, a change in the nitrate inventory of the water masses, linked to changes in nutrient supplies, stratification of the water column or ocean circulation can also alter the sedimentary δ^15^N signal. At constant primary production rates, higher input of nitrate to the surface water can result in lower δ^15^N values. Additionally, the organic matter TOC:TN (total nitrogen) ratio and δ^13^C can be used to discriminate between terrestrial, sympagic, and marine sources of sedimentary organic matter. For isotopic measurements of carbon, samples were treated with HCl followed by two distilled water rinses and were oven‐dried prior to measurements with an IRMS (IsoPrime GV). Nitrogen content and isotopic ratios were measured on untreated freeze‐dried samples with an Elemental Analyzer (Flash2000, ThermoFisher) coupled with the IRMS (Data S1). Carbon and nitrogen analyses were conducted at a 5–20 cm sampling interval, which corresponds to an effective age resolution of c. 75–440 years (mean: ~175 years).

#### 2.2.2 Diatoms

Diatoms account for an important proportion of oceanic primary production and usually dominate the arctic microplankton community during spring blooms (e.g., Lalande, Nöthig, & Fortier, 2019; Tréguer et al., 2018). Their silicified cell walls (i.e., frustules) exhibit a large morphological diversity (size, shape, level of silicification, etc.), which gives them differential sinking and sedimentary preservation potential. Their short generation time and largely species‐specific response to environmental conditions make them useful indicators of changes in sea‐surface conditions. While some diatoms thrive in the bottom layer of sea ice, a seasonal succession of taxa typically follows sea‐ice breakup leaving a unique, though fragmentary, environmental signature in the underlying sediment. Following nutrient exhaustion, fast‐growing genera, including *Chaetoceros*, can produce a rain of small, highly silicified and fast‐sinking resting spores that preserve well in the sediment and attest to past productivity levels (Abelmann, Gersonde, Cortese, Kuhn, & Smetacek, 2006). Subfossil diatom abundance and assemblage composition can thus be used to infer information about past primary production and its link to climate‐sensitive parameters, including sea ice.

Samples for diatom analyses were prepared using c. 0.3 g of dry sediment and following the standard methodology described in Crosta et al. (2020; Data S1). Quantification was done using a light microscope (Olympus BX53) with phase contrast optics, at 1000× magnification. Diatoms were analyzed at a 4 to 28 cm sampling interval, which corresponds to an effective age resolution ranging from c. 44 to 275 years (mean: ~115 years). *Chaetoceros* spores are not included in the relative abundance calculations.

Diatom concentrations were converted into fluxes by combining concentrations (valves or spores/g) with sediment densities derived from computed tomography (CT) numbers (or Hounslow units; Fortin et al., 2013) and accumulation rates derived from the age/depth model (cm/year). Since the sediment density was derived from CT numbers, fluxes are expressed as valves per unit of surface area per year, and while the trends can be compared, the flux values should not be directly compared with fluxes expressed in other units.

**PLATE 1:**
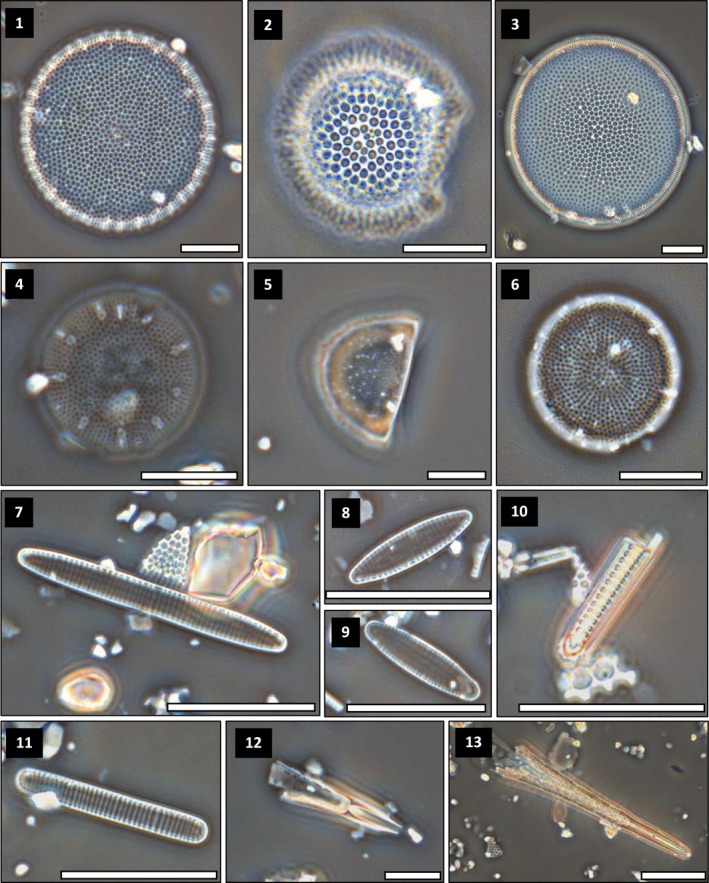
Micrographs of selected diatom taxa observed in the sediment core AMD14‐204: (1) *Thalassiosira antarctica* var. *borealis* (resting spore primary valve), (2) *T. antarctica* var. *borealis* (resting spore secondary valve), (3) *Actinocyclus curvatulus*, (4) *Thalassiosira nordenskioeldii*, (5) *Melosira arctica*, (6) *Bacterosira bathyomphala*, (7) *Fragilariopsis reginae‐jahniae*, (8) *Fragilariopsis oceanica*, (9) *Fossula arctica*, (10) fragment of *Thalassiothrix longissima*, (11) *Fragilariopsis cylindrus*, (12) fragment of *Rhizosolenia hebetata* f. *semispina*, (13) fragment of *Rhizosolenia hebetata* var. *hebetata*. Scale bar: 10 µm

*Fragilariopsis cylindrus* and *Fragilariopsis nana* were grouped together (Plate 1). The fast‐growing “marginal ice zone assemblage” is primarily represented by the cryopelagic *F. cylindrus*, *Fragilariopsis reginae‐jahniae*, *Fragilariopsis oceanica* and *Fossula arctica*. All these taxa can be found in sea ice, but generally reach their highest abundances after ice breakup (von Quillfeldt, 2004; Weckström et al., 2020). The “summer subsurface assemblage” comprises the large and highly silicified *Thalassiothrix longissima*, *Rhizosolenia* spp. (mainly *R. hebetata* f. *semispina* and *R. hebetata* var. *hebetata*) and *Coscinodiscus* spp. (notably *C. radiatu*s and *C. centralis*), whereas the “pack‐ice/drift‐ice assemblage” includes *Actinocyclus curvatulus* and *Melosira arctica* (Oksman, Juggins, Miettinen, Witkowski, & Weckström, 2019).

#### 2.2.3 Sea‐ice biomarkers

The lipid biomarkers IP_25_ and HBI III are complementary indicators of past seasonal sea ice (e.g., Belt et al., 2007, 2013; Collins et al., 2013). IP_25_ is produced by at least three pan‐Arctic diatom species (*Haslea spicula*, *Haslea kjellmanii* and *Pleurosigma stuxbergii* var. *rhomboides*; Brown, Belt, Tatarek, & Mundy, 2014, Limoges, Massé, et al., 2018) that thrive in the bottom horizon of sea ice. This source‐specific molecular compound is produced within the sea‐ice matrix during the spring bloom and deposited in seafloor sediments following ice melt. In marine settings, high‐sedimentary IP_25_ contents are typically interpreted as indicating enhanced sea‐ice concentrations, whereas the absence of IP_25_ in sediments can either reflect perennial sea‐ice cover, ice‐free conditions or insufficient production of source diatoms (e.g., Belt, 2018, 2019). HBI III is known to be biosynthesized by diatoms belonging to the genera *Rhizosolenia*, *Pleurosigma* and *Haslea* (e.g., Belt, Allard, Massé, Robert, & Rowland, 2000; Belt et al., 2017; Limoges, Massé, et al., 2018; Rowland et al., 2001). Based on its distribution in surface sediments, HBI III has been proposed as an indicator of the receding sea‐ice edge (Belt et al., 2015; Collins et al., 2013; Ribeiro et al., 2017; Smik, Belt, Lieser, Armand, & Leventer, 2016), likely reflecting the neighboring open, fresh, and stratified water of the marginal ice zone. Koch et al. (2020), however, also reported the production of HBI III in the Bering and Chukchi Sea starting during the spring under‐ice diatom bloom, although peak production was recorded in September.

Sediment samples for sea‐ice biomarker (IP_25_ and HBI III) analysis were processed according to the protocol described by Belt et al. (2007; Data S1). The data were collected using ChemStation and analyzed using MassHunter quantification software. HBIs were identified on the basis of retention time and comparison of mass spectra with authenticated standards. IP_25_ and HBI III abundances were obtained by comparison of individual GC‐MS responses against those of the internal standard and concentrations are reported in ng/g. Response factors of the internal standard versus IP_25_ were determined prior and after each analytical sequence (every 15 samples). The seasonal sea‐ice biomarkers were analyzed at a 1 to 10‐cm sampling interval, which corresponds to an effective age resolution ranging from c. 12 to 165 years (mean: ~33 years).

## 3 RESULTS

### 3.1 TOC, TN, δ^15^N and δ^13^C

**FIGURE 3:**
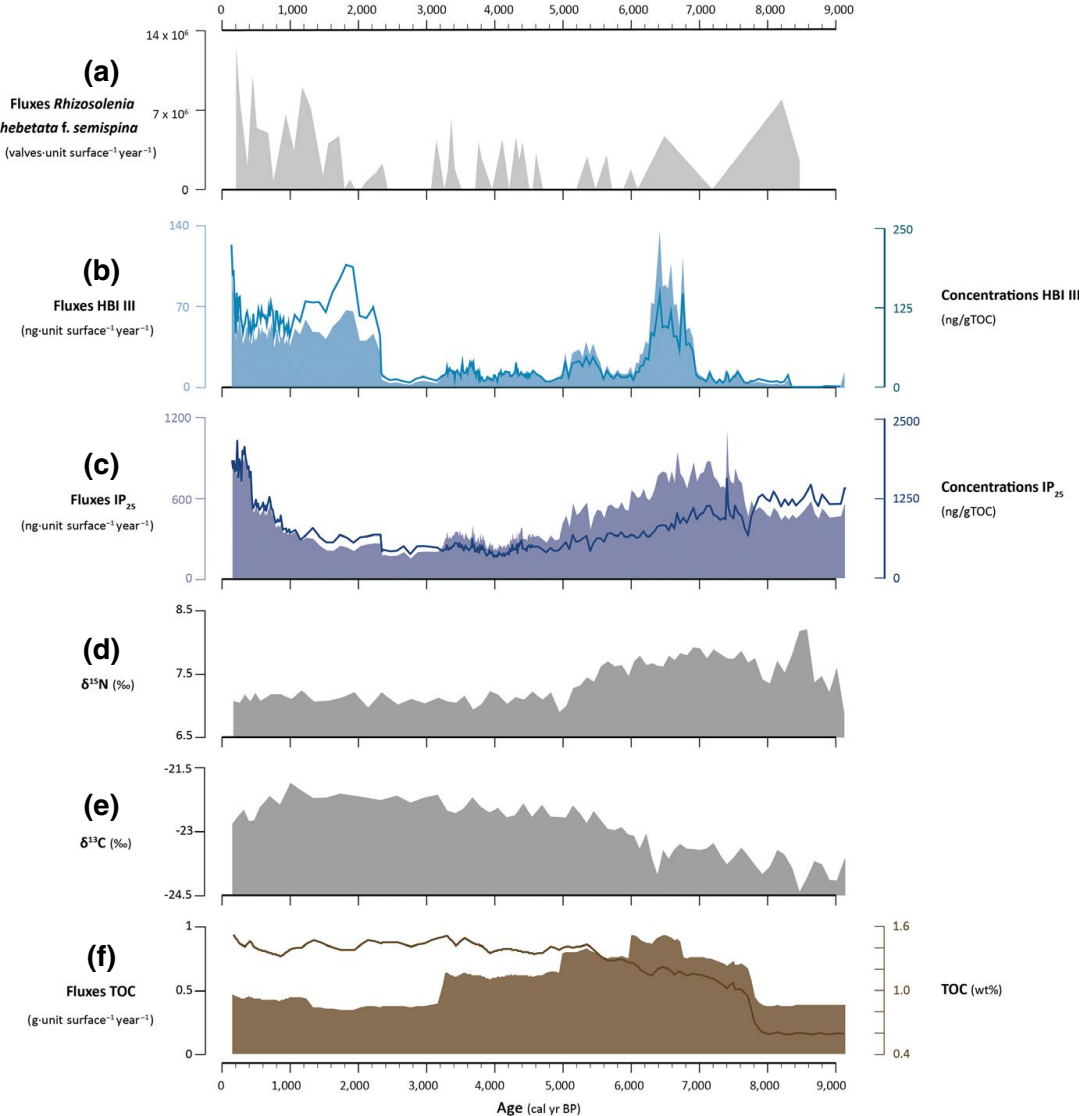
Temporal changes in selected biogenic proxies for the past c. 9,000 cal. years before present (BP). From top to bottom: (a) fluxes of the HBI III source species *Rhizosolenia hebetata* f. *semispina* (valves unit surface^−1^ year^−1^); (b) HBI III fluxes (ng unit surface^−1^ year^−1^; filled area; left *Y*‐axis), median value (dashed line; left *Y*‐axis), and absolute concentrations normalized to TOC (ng/gTOC; line; right *Y*‐axis); (c) IP_25_ fluxes (ng unit surface^−1^ year^−1^; filled area; left *Y*‐axis), median value (dashed line; left *Y*‐axis), and absolute concentrations normalized to TOC (ng/gTOC; full line; right *Y*‐axis); (d) nitrogen isotopic signature (‰); (e) carbon isotopic signature (‰); and (f) total organic carbon (TOC) fluxes (g unit surface^−1^ year^−1^; filled area; left axis) and content (wt%; full line; right *Y*‐axis)

**FIGURE 4:**
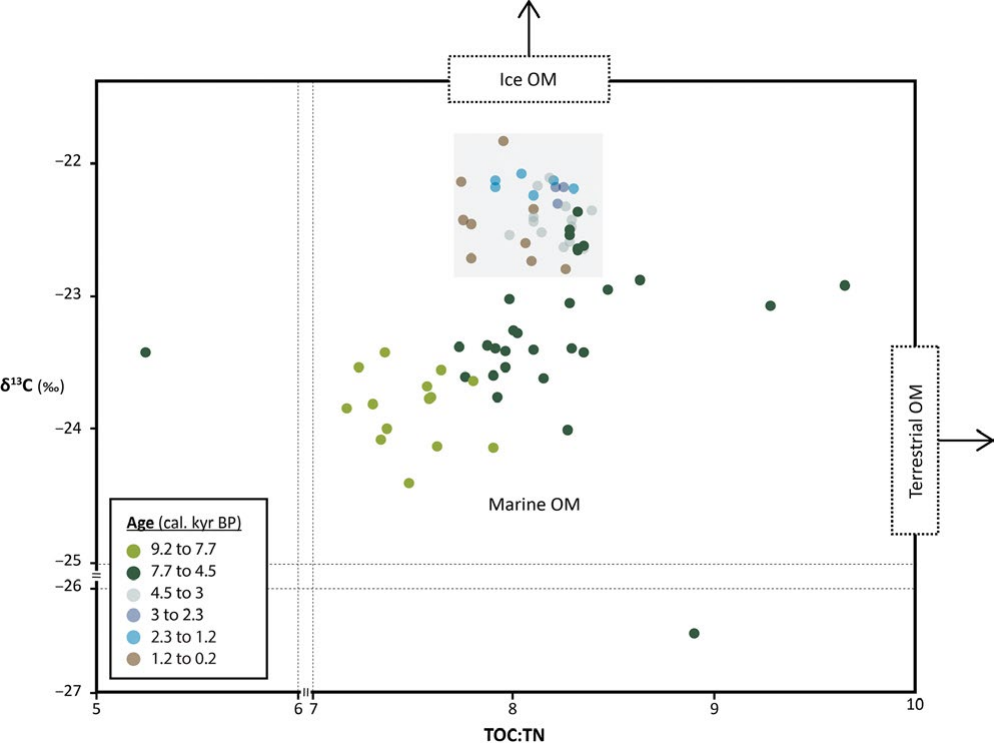
Carbon isotopic values (δ^13^C, ‰) against TOC:TN ratios of bulk organic matter. The color code indicates the age of the samples. The arrows indicate that ice particulate OM is more enriched in ^13^C compared to pelagic particulate OM values due to limited atmospheric CO_2_ exchange in the sea ice (e.g., Rau, Takahashi, Desmarais, Repeta, & Martin, 1992), and land‐derived OM yields higher TOC:TN ratios (20–100; Meyers, 1994) than aquatic OM (typically between 4 and 10, Meyers, 1994). Our data indicate a pelagic‐dominant signature, with varying mixing ratios between pelagic, sympagic, and terrestrial organic matter. In general, the elemental and isotopic signature of the organic matter shows that samples younger than 4.5 kyr BP (indicated by the gray box), and especially between 3 and 1.2 kyr BP, contain higher ratios of ice‐derived organic material

TOC contents remain low and range between 0.59 and 1.52 wt% (Data S2). The lowest values were measured in the lowermost portion of the core. They then increase sharply to c. 1 wt% around 7.7 kyr BP (Figure 3). Following this rapid increase, values continue to slowly rise before stabilizing starting around 5 kyr BP to values oscillating around 1.4 wt%. This translates into low TOC fluxes from 9.2 to 7.7 kyr BP. TOC fluxes then show a stepwise increase to reach maximum values between 6.6 and 6 kyr BP. From 6 kyr BP fluxes decrease stepwise until 3.2 kyr BP, from which point they drop to low values that are comparable to those observed between 9.2 and 7.7 kyr BP. From c. 1.4 kyr BP, TOC fluxes slowly increase toward the top of the core. TN contents vary between 0.08 and 0.18 wt%. The lowest values are also found from the bottom of the core until 7.7 kyr BP, from which point they are generally increasing. These translate into TOC:TN ratios ranging from c. 5 to 9 (Figure 4).

Nitrogen isotopic compositions range from 6.88 to 8.21‰ (Figure 3; Data S2), similar to the nitrogen isotopic signature of modern surface and subsurface nitrates in central and western Baffin Bay (Lehmann et al., 2019). δ^15^N values increase rapidly from 6.88‰ from the bottom of the core to reach a peak (8.21‰) at 8.5 kyr BP, and then decrease until 8 kyr BP. δ^15^N values subsequently rise until 7 kyr BP, from which point they decrease progressively until 5 kyr BP (Figure 3). The nitrogen composition of organic matter thereafter remains relatively constant toward the top of the core, with an average value of 7.11‰. Carbon isotopic values range from –21.89 to –24.40‰ (Figure 3; Data S2). Values are increasing from the bottom of the core, until c. 3 kyr BP when they reach a plateau and remain high (~ –22%). From 1 kyr BP, values show a general decreasing trend (Figure 3).

### 3.2 Diatoms

A total of 60 diatom taxa were identified (Data S3). Diatom concentrations range from <1 to c. 6 × 10^7^ valves/g dry sediment, which translates into fluxes between c. 0 to 4 × 10^9^ valves surface area^−1^ year^−1^ (Data S4). Our data reveal a strong temporal variability in Holocene diatom fluxes, with a 24× difference between the lowest and peak diatom fluxes.

**FIGURE 5:**
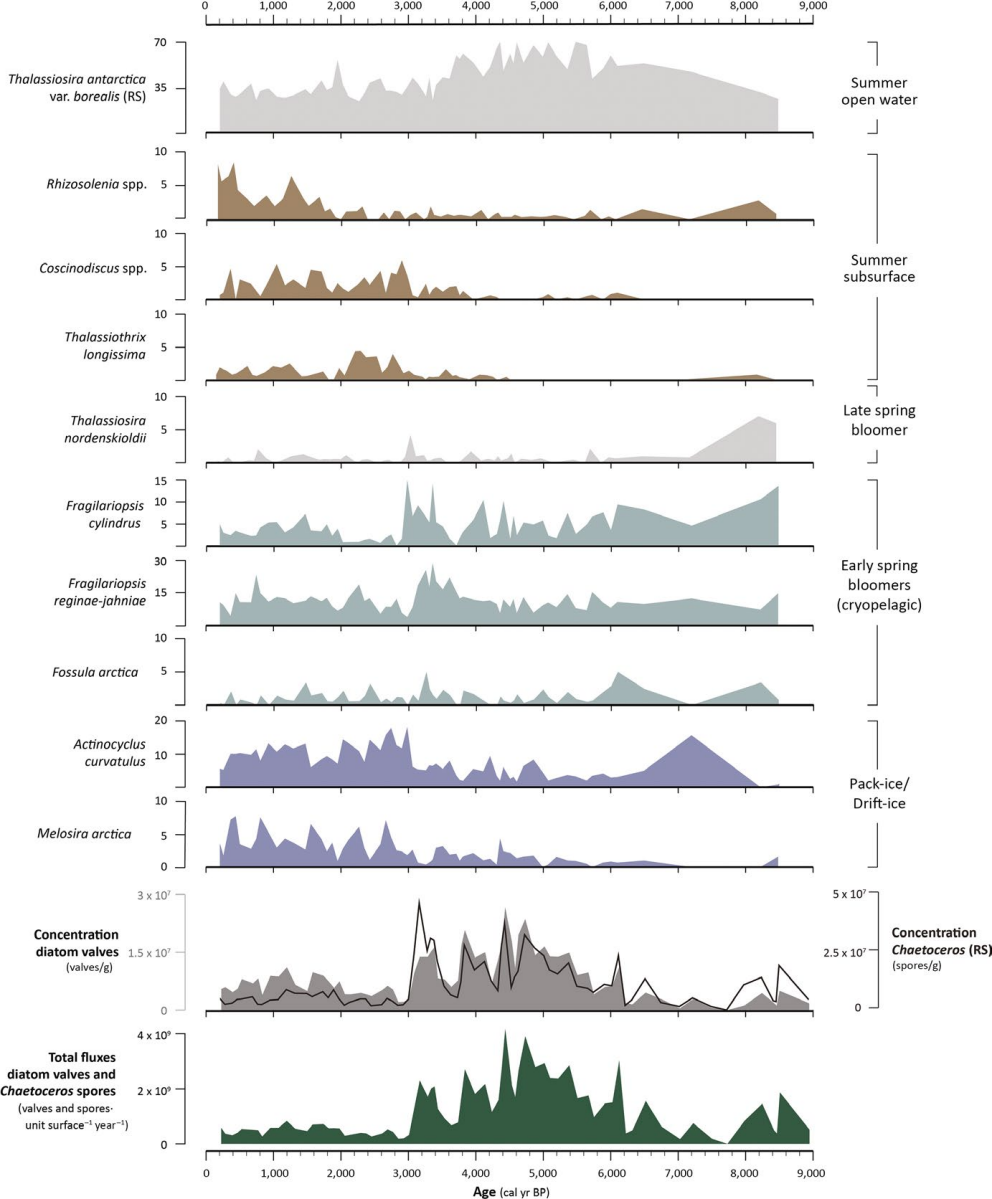
Relative abundances (%) of the main diatom taxa (excluding *Chaetoceros* resting spores), absolute concentrations (valves/g) of diatom valves (filled area) and *Chaetoceros* spp. (resting spores; line), and total diatom fluxes (valves unit surface^−1^ year^−1^; includes both diatom valves and *Chaetoceros* spores)

**FIGURE 6:**
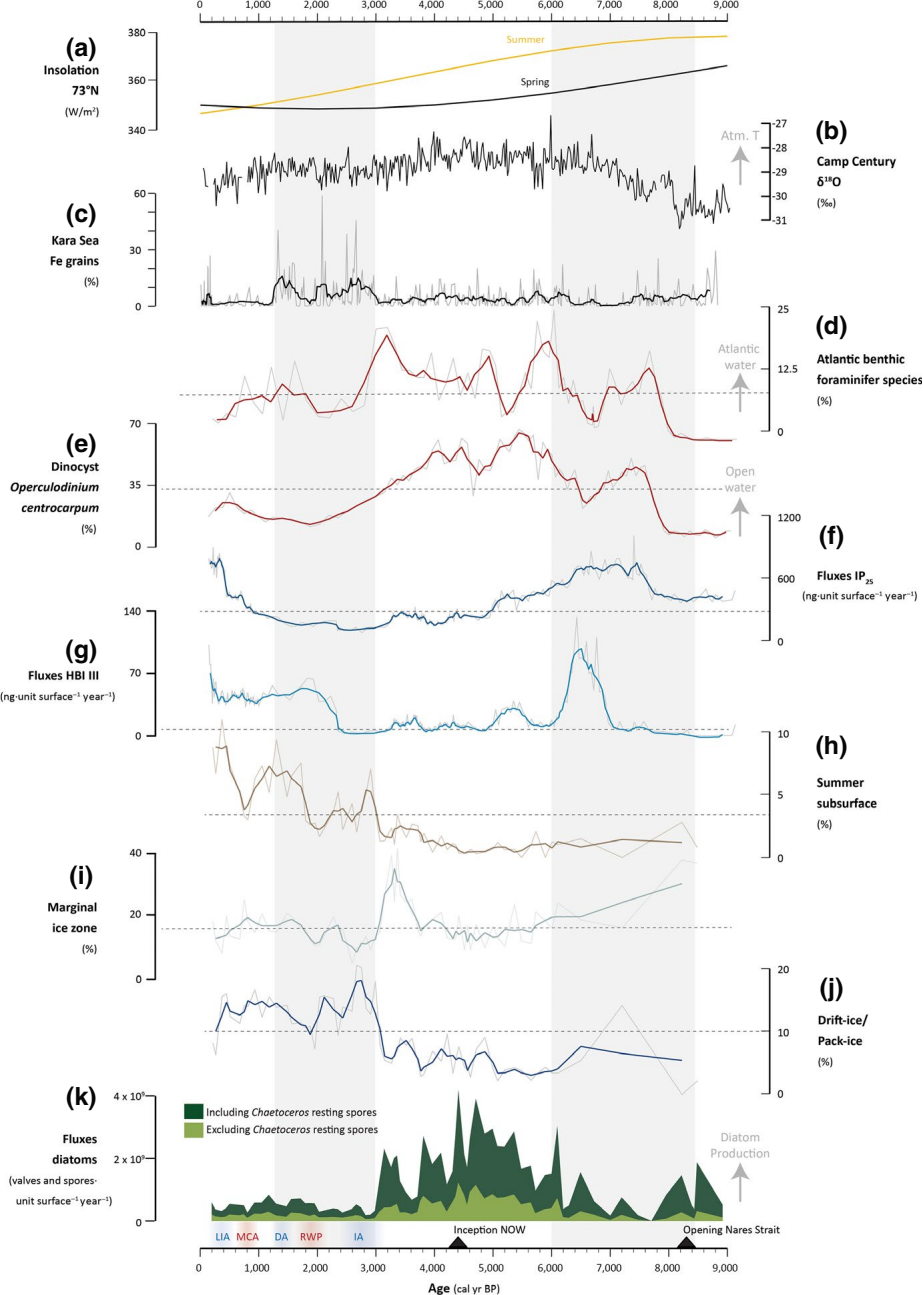
Summary figure showing primary production and sea‐ice dynamics in relation with the AO index and Holocene climate development. From top to bottom: (a) summer and spring insolation (W/m^2^) at 73°N (Laskar et al., 2004); (b) oxygen isotopic ratios (‰) at Camp Century (Vinther et al., 2009); (c) abundance of Kara Sea ice‐rafted Fe oxide grains in the Beaufort gyre (Darby et al., 2012); (d) relative contribution of the Atlantic water indicator foraminiferal group from core AMD14‐204 (%; Hansen et al., 2020); (e) relative contribution of the warm water indicator dinocyst taxon *Operculodinium centrocarpum* from core AMD14‐204 (%; Caron et al., 2019); (f) IP_25_ fluxes (ng unit surface^−1^ year^−1^; this study); (g) HBI III fluxes (ng unit surface^−1^ year^−1^; this study); (h) relative contribution of the “summer subsurface” assemblage (%; this study); (i) relative contribution of the “marginal ice zone” assemblage (%; this study); (j) relative contribution of the “drift‐ice/pack‐ice” assemblage (%; this study); and (k) total diatom fluxes (valves unit surface^−1^ year^−1^, dark green includes *Chaetoceros* spores and light green excludes *Chaetoceros* spores (this study). The gray areas indicate intervals of persistent positive AO phases (Darby et al., 2012; Funder et al., 2011). DA, Dark Age; IA, Iron Age; LIA, Little Ice Age; MCA, Medieval Climate Anomaly; RWP, Roman Warm Period

Changes in the diatom fluxes are accompanied by clear compositional shifts in the assemblages (Figure 5). The bottom of the core (9.2 to 7.7 kyr BP) is characterized by the lowest fluxes of diatom valves and moderate fluxes of *Chaetoceros* spores (Figures 5 and 6). The assemblage has a unique composition, with high contribution of the marginal ice zone group and the maximum relative abundance of *F. oceanica* (20%), *Thalassiosira nordenskioeldii* (7%), and presence of *Thalassiosira bulbosa* (<2%). Around 7.2 kyr BP, an ephemeral peak in the abundance of *A. curvatulus* is observed, while the contribution of the marginal ice zone group decreases. Total diatom fluxes remain relatively low until 6 kyr BP and then progressively increase to reach maximal values at around 4.4 kyr BP. Increasing diatom fluxes are associated with an increase in the abundance of *T. antarctica* var*. borealis* (resting spore), which largely dominates the assemblages. After 4.4 kyr BP, a stepwise decrease in total diatom fluxes is observed. At the same time, the assemblages show a decrease in the relative abundances of *T. antarctica* var*. borealis* (resting spore) and a strong increase in the marginal ice zone taxa. At 3 kyr BP, an abrupt and pronounced decline in the total diatom fluxes is accompanied by a rapid shift to a new regime of diatom productivity: a sharp decline in the marginal ice zone assemblage concurs with a marked increase in the share of the “drift‐ice/pack‐ice” and “summer subsurface” taxa. Although still a dominant contributor to the assemblages, *T. antarctica* var. *borealis* (resting spores) shows lower relative abundances from 3 kyr BP. Relatively low diatom fluxes, with slight oscillations, prevail for the rest of the core record. However, fluxes were particularly low between 3 and 2 kyr BP. From 2 kyr BP, a progressive increase in *Rhizosolenia* spp. is observed, whereas the contribution of *T. longissima*, occupying the same ecological niche, decreases to moderate values. The share of both the “drift‐ice/pack‐ice” and “summer subsurface” assemblages remains high for the rest of the core.

### 3.3 Sea‐ice biomarkers

The sea‐ice biomarker IP_25_ was detected throughout the entire record, attesting to the presence of a seasonal sea‐ice cover in the study area over the past 9.2 kyr BP. Fluxes of IP_25_ are moderately high at the bottom of the core, increase starting from 7.8 kyr BP, peak at around 7.4 kyr BP, and then progressively decrease from 6.6 until to 4.4 kyr BP (Figure 3; Data S5). A slight increase in the IP_25_ fluxes is also observed between 3.9 and 3.2 kyr BP. Between 3.2 and 1.5 kyr BP, IP_25_ reaches its lowest values. Following this interval, IP_25_ rises again to reach a maximum around 0.2 kyr BP.

The earliest peak in IP_25_ is followed by an increase in HBI III fluxes starting around 7 kyr BP. HBI III remains relatively high until 6.1 kyr BP and declines for about 600 years before increasing slightly again at 5.5 kyr BP (Figure 3; Data S5). Generally low HBI III fluxes after 5.1 kyr BP are concurrent with the decline in IP_25_. During the interval between 5.1 and 2.3 kyr BP, variations in HBI III and IP_25_ are synchronous. However, a sharp increase in HBI III at 2.3 kyr BP marks an interval of sustained higher HBI III values. HBI III increases sharply from c. 0.25 kyr BP toward the surface. Notably, the major increase in HBI III from 2.3 kyr BP is concurrent with a marked increase in the relative abundance of the HBI III‐producing species *Rhizosolenia hebetata* f. *semispina* (Belt et al., 2017; Figure 3).

## 4 DISCUSSION

### 4.1 Primary production and sea‐ice dynamics on the Northwest Greenland shelf

As illustrated by previous studies based on dinoflagellate cysts, foraminiferal assemblages, and geochemical data (Caron et al., 2019; Giraudeau et al., 2020; Hansen et al., 2020), core AMD14‐204 captures the Holocene postglacial climate development off northwest Greenland. The continuous and high‐resolution multiproxy record presented here allows us to infer detailed information about changes in seasonal sea ice and primary production.

#### 4.1.1 Low productive environment, extensive sea‐ice cover, land‐sourced meltwater influence, and deglacial opening of Nares Strait (c. 9.2–7.7 cal kyr BP)

The low organic carbon, low HBI III and moderate IP_25_ fluxes in the lowermost part of the core suggest a prolonged sea‐ice season and low productivity. Fluxes of *Chaetoceros* spores are moderate but otherwise diatom valve fluxes are much reduced (about three times below the core average). This interval notably shows a high contribution of the marginal ice zone group (Figure 6) and the highest relative abundance of *T. nordenskioeldii*. This species typically reaches its highest relative abundance after ice breakup and is thought to be a late‐spring bloomer (Oksman et al., 2019). Intermediate abundances of *T. antarctica* var. *borealis* (resting spore) additionally suggest cold, open‐water conditions during summer. The production of *Chaetoceros* spores generally represents the last stage of the diatom growth season and is initiated by nutrient exhaustion in the surface water (McQuoid & Hobson, 1996). Some *Chaetoceros* species that are lightly silicified can more easily take up nutrients and can maintain populations in nutrient‐depleted waters (Booth et al., 2002). The high δ^15^N values, along with low TOC and diatom fluxes, are coherent with a scenario of limited surface‐water nitrate availability and late sea‐ice breakup. At the closely located core GeoB19927, the IP_25_ and HBI III trends are comparable to ours, and brassicasterol and dinosterol fluxes show relatively high but decreasing values from c. 9.5 to 7.8 kyr BP (Saini et al., 2020). While our proxy signals may have been somewhat diluted by elevated clastic input during this interval, all proxies nonetheless point toward a low to moderately productive environment. This agrees with the extensive sea‐ice cover, marked meltwater and detrital sediment discharges also documented by Caron et al. (2019), and low entrainment of Atlantic water into the WGC inferred from foraminiferal assemblages (Hansen et al., 2020), during an interval of rapid thinning of the Melville sector of the Greenland Ice Sheet (Giraudeau et al., 2020). It is also between c. 9 and 8.3 kyr BP that the retreat of the Greenland and Innuitian ice sheets led to the complete opening of Nares Strait (Georgiadis et al., 2018; Jennings, Andrews, Oliver, Walczak, & Mix, 2019), establishing a new oceanic connection between the Arctic Ocean and Baffin Bay and a new source of Pacific‐derived waters into northern Baffin Bay. The increase in the δ^15^N values recorded from c. 8.4 kyr BP is coherent with the advection of isotopically heavier Pacific water into northern Baffin Bay.

#### 4.1.2 Increasing primary production, stratified water column, and onset of WGC influence (c. 7.7–6 cal kyr BP)

From 7.7 kyr BP, a steep increase in the sedimentary TOC contents indicates either a transition to a more productive system and/or reduced detrital input from meltwater. This TOC flux increase is coeval with a WGC strengthening inferred from the mineralogical and elemental composition of the sedimentary material (Caron, Montero‐Serrano, St‐Onge, & Rochon, 2020), and enhanced abundance of foraminiferal species indicative of Atlantic Water influx at our study site (Hansen et al., 2020; Figure 6). From c. 7 kyr BP, a progressive strengthening of the WGC has also been recorded in Disko Bay (e.g., Ouellet‐Bernier, de Vernal, Hillaire‐Marcel, & Moros, 2014; Perner, Moros, Jennings, Lloyd, & Knudsen, 2012) and attributed to the onset of deepwater formation in the Labrador Sea (Hillaire‐Marcel & Vernal, 2008), presumably linked to a basin‐wide reorganization of the surface circulation in the northern Atlantic following the final retreat of the Laurentide and Greenland ice sheets (Dyke & Prest, 1987; Van Nieuwenhove, Baumann, Matthiessen, Bonnet, & de Vernal, 2016; Van Nieuwenhove et al., 2018). An increase in IP_25_ fluxes accompanied by a peak contribution of *A. curvatulus*, a species strongly associated with heavy pack‐ice in northern Baffin Bay (Williams, 1986, 1990), likely further suggest an episode of heavier sea‐ice cover centered at c. 7.2 kyr BP.

Between 7 and 6 kyr BP, a clear excursion in the HBI III fluxes is coeval with a punctual increase in diatom fluxes, a sharp decline in the Atlantic Water foraminiferal species (Hansen et al., 2020; Figure 6) and a short‐lived peak in the ice‐rafted debris (IRD) content (Caron et al., 2018) at our study site. During this interval, the marked contribution of ribbon‐forming pennate *F. cylindrus* and *F. reginae‐jahniae*—two spring bloomers associated with ice melt (e.g., von Quillfeldt, 2001)—attests to cold, low‐salinity spring/early summer conditions. High dinosterol fluxes at site GeoB19927 suggest sustained high dinoflagellate productivity from c. 7.4 kyr BP (Saini et al., 2020). From 7 kyr BP Caron et al. (2019) documented a slight decrease in the dinoflagellate cyst fluxes accompanied by a decline in the phototrophic taxon *Operculodinium centrocarpum*. Diatoms and dinoflagellates have distinct life‐cycle strategies, involving different environmental and ecophysiological optima (e.g., Litchman & Klausmeier, 2008). Here, the phytoplankton signal (diatoms and dinoflagellates) may point toward a productive vernal bloom, followed by a relatively short phytoplankton growth season and/or stratified water column. In the Kane Basin, increased HBI III fluxes were recorded during this interval (core AMD14‐Kane2B, Georgiadis et al., 2020), suggesting increased mobile sea ice and freshwater outflow via Nares Strait. Coherent with the high δ^15^N and HBI III values, enhanced outflows of Arctic waters may have contributed to the already well‐stratified waters resulting from continued meltwater input from the Greenland Ice Sheet. As suggested by Hansen et al. (2020) the input of highly saline corrosive deep Arctic water or brines may have impeded the growth (or preservation) of benthic calcareous foraminifera at our study site during this interval.

#### 4.1.3 From primary production optimum with increased Atlantic signature of the WGC, to Neoglacial cooling and re‐establishment of persistent seasonal sea ice (c. 6–3 cal kyr BP)

In phase with a progressive decline in IP_25_, suggesting a transition to earlier seasonal sea‐ice retreat, diatom production rises markedly until c. 4.4 kyr BP. At core GeoB19927, this is associated with sustained high dinosterol fluxes (Saini et al., 2020). During this interval, diatom assemblages are largely dominated by the centric species *T. antarctica* var. *borealis* (resting spore), with low contributions of the marginal ice zone group (Figure 6). In modern water samples from West Greenland, the occurrence of *T. antarctica* var. *borealis* resting spores was linked to higher surface‐water temperatures, and on the Holocene timescale, based on sediment assemblages, it was linked to the influence of temperate WGC waters (e.g., Krawczyk et al., 2010, 2013; Krawczyk, Witkowski, Waniek, Wroniecki, & Harff, 2014, as *T. kushirensis*). Additionally, while diatom production progressively increases, δ^15^N values decline from the beginning of this interval until they reach a plateau at 4.8 cal kyr BP. This signal can suggest increasing nitrate supplies to the surface water due to reduced stratification. A reduced stratification would be consistent with reduced meltwater input from the Greenland Ice Sheet following the early Holocene (Giraudeau et al., 2020). Along northwest Greenland, the ice margin reached its minimum extent between ~5 and 3 kyr BP (Briner, Kaufman, Bennike, & Kosnik, 2014) and reduced meltwater runoff was inferred after 6 kyr BP (Moros et al., 2016 and references therein). Overall, this interval shows a progression to a longer duration of the open‐water growth season and surface conditions favorable to primary production, which could further be linked to the inferred generally warmer and strengthened WGC during this interval (Hansen et al., 2020; Jennings et al., 2011, 2014; Jennings, Knudsen, Hald, Hansen, & Andrews, 2002; Perner et al., 2013). It is worth noting that low amplitude peaks in HBI III were always coeval with events of reduced Atlantic benthic foraminifera species at the study site (Hansen et al., 2020), possibly indicating episodes of decreased advection of Atlantic Water, and increased water column stratification or polar inflows.

After 4.4 kyr BP, the long‐term decline in IP_25_ ceases and diatom production declines stepwise. The same declining trend is observed in the dinosterol content of core GeoB19927 (Saini et al., 2020). A gradual increase in the contribution of the marginal ice zone diatom group (Figure 6) hints at a progressive return to colder conditions, while higher δ^13^C values suggest increased contribution of ice‐derived organic matter to the sediments (Figure 4). These changes coincide with the inception of a stable and recurrent North Water polynya dated to c. 4.4 cal kyr BP (Davidson et al., 2018). This timing also coincides with a snowline lowering across 15 different ice caps in central West Greenland, which has been interpreted to reflect persistent summer cooling (Schweinsberg, Briner, Miller, Bennike, & Thomas, 2017). During the entire interval, short‐term increases in HBI III fluxes were synchronous with low amplitude rises in IP_25_ fluxes, suggesting that the orbitally driven long‐term Neoglacial cooling (Vinther et al., 2009) associated with decreasing summer insolation in the Northern Hemisphere was interrupted by short events of fluctuating sea‐ice conditions at the study site. Starting around 3.5 kyr BP, the major increase in the contribution of the marginal ice zone group, and especially *F. reginae‐jahniae*, suggests the re‐establishment of more persistent seasonal sea ice (Weckström et al., 2020; Figure 6). Interestingly, this is synchronous with an increase in the dinoflagellate productivity reported at core GeoB19927 (Saini et al., 2020), and marked increase in the dinoflagellate cyst fluxes, notably involving a decline in *O. centrocarpum* and steady increase in the spring bloomer *Pentapharsodinium dalei* at our study site (Caron et al., 2019). Such high contribution of the marginal ice zone diatom assemblage, high abundance of the Atlantic benthic foraminiferal species, and high fluxes of dinosterols and dinoflagellate cysts in the study area may indicate a change in sea‐ice seasonality and an increased proximity of the study site to the spring marginal ice edge.

#### 4.1.4 Primary production decline, extensive sea‐ice cover, stratified water column, and weakened Atlantic water signature of the WGC (c. 3.0–1.2 cal kyr BP)

We record an abrupt change in the diatom regime taking place at 3 kyr BP. IP_25_, HBI III, and diatom fluxes (including *Chaetoceros* spores) decline to minimum values at the beginning of this interval. A steep decline in the contribution of the marginal ice zone group is shortly followed by a pronounced increase in the “drift‐ice/pack‐ice” and “summer subsurface” assemblages (Figure 6). The drift‐ice/pack‐ice assemblage includes *M. arctica*, a species known to form dense aggregates under sea ice, which can be abundant under drift ice and has been observed to form free‐floating filaments in the meltwater layer of the Arctic Ocean (Poulin, Underwood, & Michel, 2014; von Quillfeldt, Ambrose, & Clough, 2003). It secretes gelatinous extracellular polysaccharide substances that facilitate its anchoring to the ice matrix (Krembs, Eicken, & Deming, 2011) and has been linked with widespread spore formation following nutrient depletion and grazing activities toward the end of the spring bloom in Baffin Bay (Lafond et al., 2019). In surface sediments and sediment traps from Greenland, *M. arctica* is strongly associated with spring/summer sea ice (Krawczyk et al., 2017; Luostarinen et al., 2020). The drift‐ice/pack‐ice assemblage also includes *A. curvatulus*, a cold‐water species that is associated with high sea‐ice concentrations (Oksman et al., 2019) and heavy pack ice in northern Baffin Bay (Williams, 1986, 1990). The summer subsurface assemblage includes the genus *Coscinodiscus,* containing species known to thrive at low light irradiance levels under pack ice (Duerksen et al., 2014; Kemp, Pike, Pearce, & Lange, 2000), and contributing substantially to the early spring bloom in the North Water polynya (Lovejoy, Legendre, Martineau, Bâcle, & von Quillfeldt, 2002). Outside of the polar regions, *Coscinodiscus* spp. (such as the dominant *Coscinodiscus* species in our study) are an important constituent of the subsurface chlorophyll maximum (Odebrecht & Djurfeldt, 1996; Weston, Fernand, Mills, Delahunty, & Brown, 2005). This assemblage also comprises *Rhizosolenia* sp. and *T. longissima*. Both Rhizosolenids and *Thalassiothrix* spp. have been documented to thrive in the zones of converging surface currents: their capacity to regulate buoyancy, exploit deep nutrient sources and reproduce at low light conditions, make them adapted to a stratified water column (e.g., Kemp et al., 2006 and references therein). *T. longissima*, which yielded its maximum abundance (~4%) during this interval, is further associated with relatively warm summer open‐water conditions (Knudsen et al., 2008; Oksman et al., 2019), and marine to brackish conditions (Pearce, Weckström, Sha, Miettinen, & Seidenkrantz, 2014). Albeit distinct from the rest of the core, this diatom assemblage has a strong sea‐ice fingerprint.

Between 3.2 and 2.3 kyr BP, the sharp decline in primary production inferred here concurs with significant worldwide glacier advances (e.g., Grove, 2004; Wanner et al., 2008). Regionally, it is concomitant with inferred climate deterioration in northern Baffin Bay (Knudsen et al., 2008; Levac et al., 2001; St‐Onge & St‐Onge, 2014), marked reduction in the meltwater discharge at 3.2 kyr BP in southwestern Greenland fjords (Møller et al., 2006; Seidenkrantz et al., 2007), a major temperature drop in Jakobshavn Isbræ at c. 3 kyr (Young & Briner, 2015) and shift toward cooler conditions in Disko Bay from 3.5 to 2.6 kyr BP (Moros et al., 2016). Additionally, Thomas, Briner, Ryan‐Henry, and Huang (2016) reported a steady decrease in winter snowfall in western Greenland from 4.4 to 2 kyr BP, implying a more extensive sea‐ice cover that limited not only moisture availability but also primary production during this interval. In this context, our low IP_25_ flux values appear puzzling. A similar low IP_25_ signal from the neighboring core GeoB19927 has been interpreted to reflect low sea‐ice concentrations under the influence of a persistent WGC (Saini et al., 2020). However, this interpretation is difficult to reconcile with the overall low primary production, relatively high organic matter δ^13^C, sudden decrease in the contribution of planktonic and benthic Atlantic Water foraminiferal species at the study site (Hansen et al., 2020), and low dinocyst fluxes dominated by cysts of the spring bloomer *P. dalei* (Caron et al., 2019). Our proxy record suggests that conditions other than sea‐ice presence may have affected the growth of IP_25_‐producing species or IP_25_ biosynthesis during this interval. Changes in nutrient availability can exert a major control on total primary production and influence the cellular HBI synthesis in diatoms (Brown et al., 2020). Considering that the assemblages are composed of large diatoms that typically benefit from higher nutrient levels (i.e., larger diffusion boundary layer), the hypothesis of a decrease in the nutrient availability is unlikely. Alternatively, a change in the ice matrix linked to reduced sea‐surface salinity may have impacted the settlement efficiency and growth of IP_25_‐producing sympagic algae (Gosselin, Legendre, Therriault, Demers, & Rochet, 1986; Poulin, Cardinal, & Legendre, 1983) as seen in coastal sediments and sea ice from Greenland fjords (Limoges, Massé, et al., 2018; Limoges, Ribeiro, et al., 2018; Ribeiro et al., 2017). This could partly explain the presence of sub‐ice colonial species such as *M. arctica* that have developed strategies to mitigate the effect of unfavorable sea‐ice microstructure (Krembs et al., 2011). To the north, evidence of instabilities in the Kane Basin ice arch (Georgiadis et al., 2020) may suggest progressively increasing outflow of freshwater into northern Baffin Bay from 3 kyr BP. As also raised by Hansen et al. (2020), an increased polar influence was documented in other cores from the Labrador shelf (Gibb, Steinhauer, Fréchette, de Vernal, & Hillaire‐Marcel, 2015; Lotche et al., 2019). The observed shift to giant and highly silicified diatom taxa observed here would further support increased input of Pacific waters high in nutrients (especially Si and P) and/or decreased upstream nutrient uptake. Our data caution for the use of sedimentary IP_25_ as a sole indicator for past fluctuations in sea ice and suggest that ice‐laden conditions persisted into the growing season of diatoms despite low sedimentary IP_25_ values. Furthermore, we argue that the drastic changes in the diatom species composition and production reflect an important oceanographic reorganization during this interval (see Section 4.2).

From 2.3 to 1.2 kyr BP, HBI III increases sharply and the diatom assemblages show a progressive increase in *Rhizosolenia* spp. and *F. cylindrus*, and a decrease in *T. longissima*. The proxy signal suggests enhanced surface‐water cooling and freshening during this interval, likely partly associated with increased meltwater input. This is supported by the observed increase in IRD between 2.3 and 2 kyr BP at our study site (Caron et al., 2020), and is coherent with the retreat of the Qangattaq ice cap on the Nuussuaq peninsula between c. 2.5 and 1.9 kyr BP (Schweinsberg et al., 2017) and the Greenland ice‐sheet margin in Northern Nunatarssuaq between 2.1 and 1.6 kyr BP (Farnsworth et al., 2018), at the transition to a period of significant boreal atmospheric warming that is commonly referred to as the Roman Warm Period (~2.0–1.65 kyr BP, Wanner et al., 2008). Biomarker and foraminiferal datasets further suggest the potential complete collapse of the Kane Basin ice arch in Nares Strait during this interval (Georgiadis et al., 2020), which would have further promoted the export of freshwater and (multiyear) ice into northern Baffin Bay.

#### 4.1.5 Low primary production, extensive but fluctuating sea‐ice conditions (c. 1.2–0.2 cal kyr BP)

The last 1.2 kyr BP were characterized by a marked increase in IP_25_ and generally stable HBI III fluxes, high contribution of the pack‐ice/drift‐ice and summer subsurface assemblages, moderate contribution of the marginal ice zone assemblage, and relatively high organic matter δ^13^C values, suggesting the presence of an overall extensive, but fluctuating sea‐ice cover (Figures 4, 5, 6). While diatom fluxes remain low for the complete interval, the signal is overprinted by high frequency events of low amplitude increase and decrease in diatom production. Saini et al. (2020) also reported low brassicasterol and dinosterol fluxes. Collectively, the diatom, brassicasterol, and dinosterol signals contrast with the high primary productivity reconstructed using the dinoflagellate cyst‐based modern analogue technique (MAT; Caron et al., 2019). We hypothesize that the MAT signal is steered by the dominance (50%–60%) of cysts of the phototrophic species *P. dalei*, which often occurs in large numbers in the productive waters of the early season bloom, but whose intense, transient pulses of cyst production rather seem to be favored by the highly stratified waters resulting from ice melt (e.g., Heikkilä et al., 2014; Howe, Austin, Forwick, & Paetzel, 2010). During this interval, low primary production would have resulted from a relatively extensive seasonal sea‐ice cover and comparatively short open‐water season during an interval of generally cold atmospheric temperatures (Lasher et al., 2017; Lecavalier et al., 2017). A re‐advance of the Upernavik Isstrøm was dated to c. 0.6 kyr BP (Briner, Hakansson, & Bennike, 2013). At the top of the core, the sharp increase in HBI III and *Rhizosolenia* spp. hint at a possible return to more stratified conditions and increased freshwater influence.

### 4.2 Impact of millennial‐scale AO variability on primary production

Millennial and centennial variations in the dominant atmospheric mode have been identified in various studies. These are based on, to name a few, indicators of sea‐ice drift patterns in the Arctic Ocean, such as ice‐rafted iron in the Beaufort Sea (Darby et al., 2012) and the abundance and origin of driftwood on the Northern Greenland shores (Funder et al., 2011), as well as tracers of exceptional rainfall events in North American lakes (Noren, Bierman, Steig, Lini, & Southon, 2002). While a dominantly positive AO phase was suggested for the northern Greenland Holocene Thermal Maximum between 8.5 and 6 kyr BP (Funder et al., 2011), it is only by ~6.8 kyr BP that modern‐type ocean surface circulation presumably was established in the North Atlantic (Van Nieuwenhove et al., 2018). Given this and the lower resolving power of the diatom analyses (due to low diatom valve concentrations) in the lower part of our core (9.2 to 6 kyr BP), we refrain from linking our proxy record to a predominant atmospheric circulation pattern during this interval. We however reconstruct moderate diatom production associated with increased Arctic water and drift‐ice influence, and a stratified water column. From 6 kyr BP and as solar irradiance decreases, we note that the onset of a 3,000‐year phase of dominantly negative AO (Darby et al., 2012; Funder et al., 2011; Staines‐Urías et al., 2013) is associated with sustained higher diatom fluxes and abundance of Atlantic benthic foraminiferal taxa. The proxy signal suggests a longer duration of the open‐water growth season and generally warmer and strengthened WGC during this interval. The initial phase of the Neoglacial cooling was then marked by the establishment of progressively more extensive spring sea ice, which translated into a stepwise overall decrease in primary production starting at 4.4 kyr BP and sharp increase in the dominance of spring marginal ice zone taxa.

**FIGURE 7:**
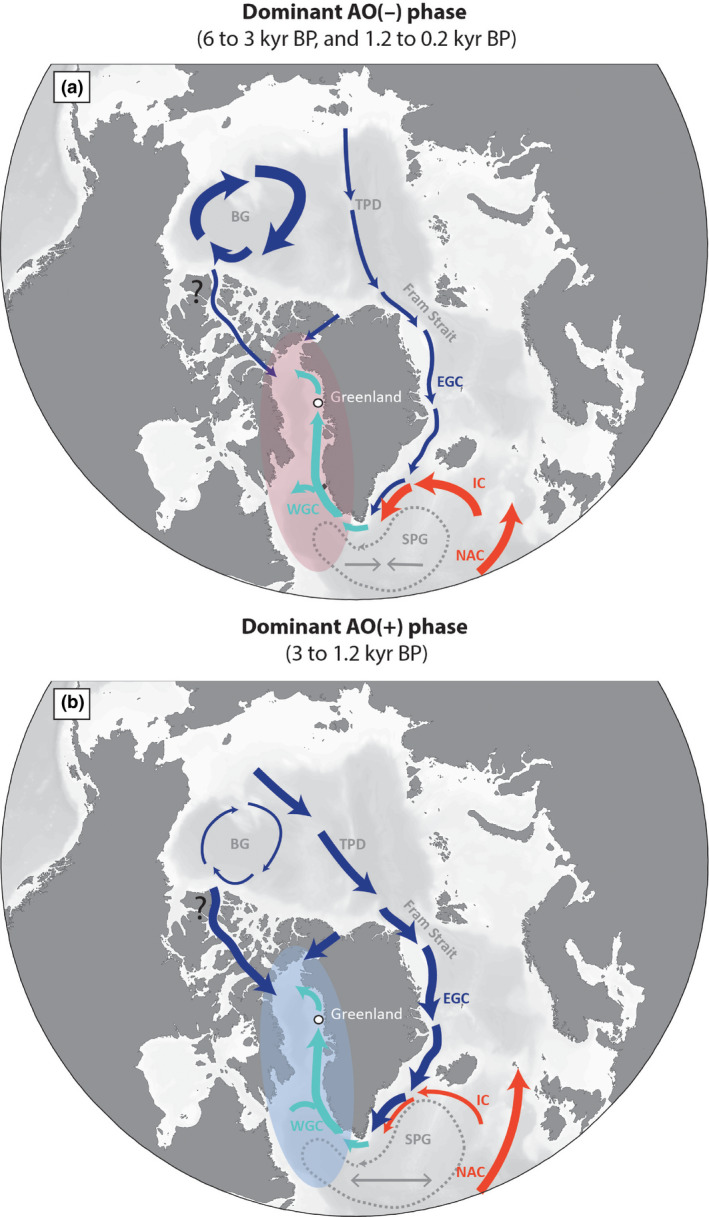
Simplified schematic representation of surface circulation under persistent low and high AO polarity index. The areas colored in red and blue reflect the milder and colder atmospheric winter temperatures during negative and positive AO phases, respectively. (a) During AO(−), a strong Beaufort gyre promotes the formation of multiyear sea ice and is linked to generally reduced export of freshwater and ice from the Arctic Ocean via Fram Strait (Rigor et al., 2002). The transpolar drift stream follows the Lomonosov Ridge (Steele et al., 2004; Talley, Pickard, Emery, & Swift, 2011). The East Greenland Current, an extension of the transpolar drift, is relatively weaker. Multiyear ice and weaker winds may facilitate the formation of ice bridges in Nares Strait, restricting export fluxes through this gateway (Georgiadis et al., 2020). In the North Atlantic, a relatively contracted subpolar gyre facilitates the entrainment of warm and salty Irminger waters into the WGC (e.g., Morley et al., 2014; Sarafanov, 2009). (b) During AO(+) a weaker Beaufort gyre is associated with thinner Arctic Ocean sea ice, especially in the eastern sector (Rigor et al., 2002), which enhances sea‐ice mobility and favors the export of solid and liquid freshwater via the main Arctic gateways. The transpolar drift stream flows nearly directly from the Bering Strait to the northern side of Greenland (Steele et al., 2004). This translates into increased EGC strength. Thinner Arctic sea ice and strong winds may contribute to weaken the Nares Strait ice bridges (Georgiadis et al., 2020), promoting freshwater and drift‐ice exports through this gateway. Export across the Canadian Arctic Archipelago channels is mainly in the form of liquid freshwater (Serreze et al., 2006). Atmospheric temperatures over Labrador, Greenland, and the western subpolar North Atlantic decrease (Talley et al., 2011). AO(+) is associated with strong westerlies, cold sea‐surface temperatures in the Labrador Sea and subpolar gyre, and expansion of the subpolar gyre, with consequent decreased northwestward entrainment of Atlantic waters, which are confined to the easternmost part of the North Atlantic (e.g., Morley et al., 2014; Sarafanov, 2009). BG, Beaufort Gyre; EGC, East Greenland current; IC, Irminger Current; SPG, Subpolar Gyre; TPD, Transpolar drift; WGC, West Greenland Current

The diatom productivity collapse documented at 3 kyr BP is coeval with fluctuations reported from several other areas of the northern North Atlantic region (e.g., Douarin et al., 2016; Jennings et al., 2011; Rasmussen, Thomsen, Troelstra, Kuijpers, & Prins, 2002), implying a large‐scale oceanographic reorganization. This important cooling event was further associated with a slowdown of the Atlantic meridional overturning circulation around 3.1–2.4 kyr BP (Oppo, McManus, & Cullen, 2003). As summarized by Morley et al. (2014), this shift is thought to be partly associated with a strengthened EGC provoking a southeastward movement of the subarctic front and longitudinal stretching of the subpolar gyre. This cooling event coincides with a swing from persistently negative to positive AO at c. 3 kyr BP (Darby et al., 2012; Funder et al., 2011; Figure 6), reinforcing the hypothesis that millennial‐scale AO‐driven changes in ocean conditions may have prompted changes in sea ice and primary production off northwest Greenland during the mid‐to‐late Holocene. While positive winter AO is typically associated with colder than normal atmospheric temperatures in West Greenland (Box, 2002), the long‐term decreased contribution of the warm Irminger Current into the WGC (e.g., Andrews & Jennings, 2014; Flatau et al., 2003; Morley et al., 2014; Sarafanov, 2009) may have exacerbated atmospheric cooling along the West Greenland margin from 3 kyr BP (Figure 7). Such a reduction of Atlantic‐sourced water to the WGC from 3 to 2.3 kyr BP would have contributed to later sea‐ice melt. This hypothesis is supported by the major decline in both the Atlantic water foraminiferal species (Hansen et al., 2020) and the relatively warm sea‐surface indicator dinoflagellate cyst *O. centrocarpum* (Caron et al., 2019) at our study site. To the north, evidence of instabilities in the Kane Basin ice bridge from 3 kyr BP (Georgiadis et al., 2020) also suggests progressively increasing outflow of polar freshwater into northern Baffin Bay. Increased input of nutrient‐rich (particularly Si) polar water would have promoted the large and highly silicified diatom flora while also contributing to a stratified upper water column. The resulting ice‐laden and stratified waters, and short growth season would have led to the major decline in primary production.

At 2.3–2 kyr BP, Darby et al. 2012) inferred an episode of unprecedented high positive AO for the Holocene (Figure 6), which coincides with a major increase of HBI III at our study site. During this episode, the lowest sea‐ice concentrations were inferred at Kane Basin, suggesting that the Kane Basin ice bridge was much weakened (Georgiadis et al., 2020), presumably impacting the functioning of the North Water polynya. While no modern analog exists for an AO event of this amplitude, we hypothesize that it may have resulted in important exports of drift ice and polar waters via Nares Strait. It is further notable that this event shortly preceded the onset of the Roman Warm Period, which was coeval with a return to weaker AO (Darby et al., 2012), decreased polar water influence in the subpolar North Atlantic (Moffa‐Sánchez & Hall, 2017) and slightly increased productivity at the study site. Our proxy record suggests that this connectivity between persistent AO phase and large‐scale oceanography has also prevailed during the late Holocene and, superimposed on radiative changes, likely played a key role in the Baffin Bay oceanography, and in the onset and length of the open‐water growth season along the West Greenland margin, with consequences for microalgal production and phenology.

### 4.3 Future evolution of marine primary production

Our proxy record suggests that over the last 9,000 years, modulations in millennial‐scale AO modes have been linked to the variability in sea ice and diatom production on the northwest Greenland shelf by exerting a control on the physicochemical conditions of the water column (e.g., thermal stratification, glacial meltwater input, nutrient supply, sea‐ice regime). Since it is generally assumed that climate warming in the Arctic will cause an earlier vernal bloom and extend the open‐water growth season, it can be tempting to predict a future increase in primary production, similar to that observed between 6 and 4.4 cal kyr BP in our study area. During this interval, phytoplankton production (diatoms and dinoflagellates) appears to have been promoted by the enhanced entrainment of Atlantic water by the WGC and earlier sea‐ice breakup during a predominantly negative AO phase. However, since studies suggest that the Arctic warming and the Arctic Amplification (e.g., Manabe & Wetherald, 1975; Serreze, Barrett, Stroeve, Kindig, & Holland, 2009) may favor positive‐like AO conditions, notably associated with a wavier flow of tropospheric circulation and more frequent extreme winter weather conditions in the North Hemisphere mid‐latitudes (Cohen, Pfeiffer, & Francis, 2018), neither the highly productive interval from 6 to 4.4 cal kyr BP, nor any other Holocene interval can be regarded as a direct analog for the future. Instead, earlier and rapid seasonal sea‐ice melt may affect the timing and production of fast‐growing sea ice‐associated taxa (e.g., Tedesco, Vichi, & Scoccimarro, 2019). Additionally, our long‐term record clearly shows that thermal and freshwater‐induced upper water column stratification resulting from atmospheric warming, increased glacial meltwater input and polar water/drift‐ice throughflows such as those taking place during positive AO intervals and predicted to increase in a warming Arctic, have a strong impact on community structure and are generally detrimental to diatom productivity. Hence, future diatom productivity in the area will be modulated by the balance between the effects of a longer open‐water growth season versus the effects of an enhanced water column stratification. This will certainly result in changes in microalgal community structure and phenology.

## 5 CONCLUSIONS

Our multiproxy record supports the hypothesis of a pervasive effect of the dominant AO phase on sea‐ice conditions and diatom production on the northwest Greenland shelf. Most notably, we show that an important and rapid decline in primary production starting around 3 cal kyr BP was coeval with a shift from low to high AO polarity values.

Changes in sea ice and diatom productivity over the past 9,000 years on the northwest Greenland shelf can be summarized as follows:

1. Extensive seasonal sea‐ice cover from 9.2 to 7.7 cal kyr BP associated with low to moderate diatom production during an interval of significant meltwater influence and deglacial opening of the Nares Strait gateway.
2. Polar water outflow event centered at c. 6.6 cal kyr BP associated with increased HBI III fluxes and a moderately high diatom production.
3. Progressive decline in seasonal sea‐ice concentrations from 6 cal kyr BP culminating with primary production optimum.
4. Onset of the Neoglacial cooling at c. 4.4 cal kyr BP accompanied by progressively increasing sea‐ice concentrations and decreasing phytoplankton production.
5. Major shift in diatom productivity at 3 cal kyr BP reflecting a major oceanographic reorganization presumably related to a swing from dominantly negative to positive AO. Resulting decrease in the Atlantic‐sourced influence of the WGC and increase in Arctic throughflows likely led to a shorter open‐water growth season and generally reduced primary production. These conditions were exacerbated from 2.3 to 2 cal kyr BP during an interval of record‐high positive AO.
6. From 1.2 to 0.2 cal kyr BP, only slight oscillations in diatom production and generally extensive seasonal sea‐ice cover occurred during an interval of dominantly negative AO.

## Supporting information

Supplementary Material

Supplementary Material

Supplementary Material

Supplementary Material

Supplementary Material

## Data Availability

The data that supports the findings of this study are available in the supplementary material of this article. Subfossil diatom counts are also available from the corresponding author upon request.
