# Supplementary material for: Learning from the past: Impact of the Arctic Oscillation on sea ice and marine productivity off northwest Greenland over the last 9,000 years
Source: Glob Chang Biol. 2020 Oct 13;26(12):6767–86. doi: 10.1111/gcb.15334 (PMC7756419; doi:10.1111/gcb.15334)
Supplement: Supplementary file 1 — Supplementary Material [file GCB-26-6767-s001.docx]

**S1. Additional information material and methods**

**Total organic carbon and nitrogen isotopic analysis**

Nitrogen isotopic ratios were measured on untreated freeze-dried samples with the IRMS. Daily drift of the IRMS was monitored with standard measurements on casein, glycine and acetanilide. The isotopic ratios are expressed in notation as deviations per mil (‰), so that δ_sample_ = 1000 x (R_sample_/R_standard_ — 1), where R is the ratio of heavy to light isotope (^15^N/^14^N; ^13^C/^12^C).

**Diatoms**

To remove the organic material, the sediment was oxidized at 75°C for 6 hours using a solution of hydrogen peroxide and tetrasodium pyrophosphate. A few drops of HCl (10%) were added to dissolve carbonates. Samples were then rinsed four times with distilled water through centrifugation. A few drops of ammonia were added after the final centrifugation cycle to remove clay from the suspension. A known volume of the final suspension was added to a petri dish, allowed to dry on a coverslip and subsequently mounted in Naphrax^TM^ for observation.

**Sea-ice biomarkers**

Prior to analytical treatment, an internal standard (7-hexylnonadecane) was added to ∼0.5 g of the freeze-dried and homogenized sediment samples. Total lipids were ultrasonically extracted (3 times) using a mixture of dichloromethane (DCM: CH_2_Cl_2_) and methanol (MeOH) (2:1, v/v). Extracts were pooled together, and the solvent was removed by evaporation under a slow stream of nitrogen. The total extract was subsequently suspended in hexane and purified through open column chromatography (SiO_2_). HBIs were eluted using hexane (8mL). Procedural blanks and standard sediments were analyzed every 15 samples. Hydrocarbon fractions were analyzed using an Agilent 7890 gas chromatograph (GC) fitted with 30m fused silica Agilent J&C GC columns (0.25mm internal diameter and 0.25μm phase thickness) and coupled to an Agilent 5975C Series mass selective detector. Oven temperatures were programmed as follows: 40–300°C at 10°C min^−1^, followed by an isothermal interval at 300°C for 10min.

**S2.** TOC, TN, δ^15^N and δ^13^C data

**S4.** Diatom data

**S5.** HBI data

**S6.** Holocene changes in **A)** HBI III concentrations (ng g^-1^), and **B)** IP_25_ concentrations (ng g^-1^).

**
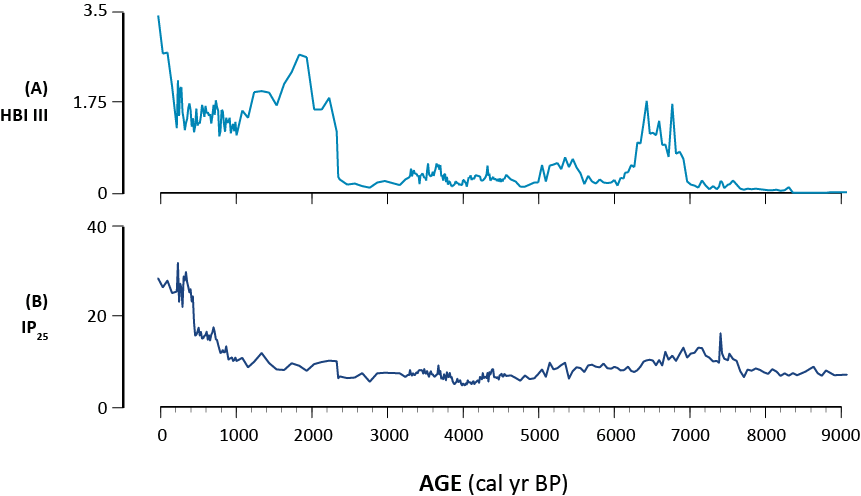
**
