# Supplementary material for: Learning from the past: Impact of the Arctic Oscillation on sea ice and marine productivity off northwest Greenland over the last 9,000 years
Source: Glob Chang Biol. 2020 Oct 13;26(12):6767–86. doi: 10.1111/gcb.15334 (PMC7756419; doi:10.1111/gcb.15334)
Supplement: Supplementary file 3 — Supplementary Material [file GCB-26-6767-s003.docx]

**S3.** List of diatom taxa encountered in core AMD14-204.

| **CENTRIC DIATOMS** | **PENNATE DIATOMS** |
| --- | --- |
| *Achnantes groenlandica* (Cleve) Grunow | *Cocconeis californica Grunow* |
| *Actinocyclus curvatulus* Janisch | *Cocconeis costata* Gregory |
| *Asteromphalus* spp. | *Cocconeis scutellum* Ehrenberg |
| *Bacterosira bathyomphala* Cleve | *Cocconeis* spp. |
| *Bacterosira bathyomphala* (rs) Cleve | *Diploneis* spp. |
| Centric spp. | *Fossula arctica* Hasle, Syvertsen & von Quillfeldt |
| *Chaetoceros affinis* (rs) | *Fragilariopsis atlantica* Paasche |
| *Chaetoceros debilis* (rs) | *Fragilariopsis cylindrus* (Grunow) Krieger in Helmscke & Krieger |
| *Chaetoceros diadema* (rs) | *Fragilariopsis nana* (Steemann Nielsen) Paasche |
| *Chaetoceros radicans* (rs) | *Fragilariopsis oceanica* (Cleve) Hasle |
| *Chaetoceros* spp. (rs) | *Fragilariopsis reginae-jahniae* Witkoswki, Lange-Bertalot & Metzeltin |
| *Coscinodiscus centralis* Ehrenberg | *Fragilariopsis* spp. |
| *Coscinodiscus radiatus* Ehrenberg | *Gomphonemopsis littoralis* (Hendey) Medlin |
| *Coscinodiscus* spp. | *Grammatophora angulosa* var. *islandica* |
| *Cymatotheca* spp. | *Navicula* spp. |
| *Melosira arctica* Dickie | *Nitzschia* spp. |
| *Melosira setosa* Greville | *Pauliella taeniata* (Grunow) Round & Basson |
| *Odontella aurita* (Lyngbye) Agardh | Pennate spp. |
| *Paralia sulcata* (Ehrenberg) Cleve | *Pseudogomphonema* (Grunow) Medlin cf. *kamtschaticum* |
| *Podosira* cf. *stelligera* | *Synedropsis recta* Hasle, Medlin & Syvertsen |
| *Porosira glacialis* (Grunow) Jørgensen | *Tabulata tabularia* (C. Agardh) Snoeijs |
| *Rhizosolenia hebetata* f. *semispina* (Hensen) Gran | *Thalassionthrix longissima* Cleve & Grunow |
| *Rhizosolenia hebetata* Bailey f. *hebetata* |  |
| *Rhizosolenia* spp. |  |
| *Shionodiscus oestrupii* (Ostenfeld) Alverson, Kang et Theriot |  |
| *Shionodiscus trifultus* (G. Fryxell) Alverson, Kang et Theriot |  |
| *Thalassiosira anguste-lineata* (A. Schmidt) G. Fryxell & Hasle |  |
| *Thalassiosira antarctica* var. *borealis* Fryxell, Doucette & Hubbard |  |
| *Thalassiosira antarctica* Comber var. *borealis* (rs) |  |
| *Thalassiosira bulbosa* Syvertsen |  |
| *Thalassiosira constricta* Gaarder |  |
| *Thalassiosira eccentrica* (Ehrenberg) Cleve |  |
| *Thalassiosira gravida* Cleve |  |
| *Thalassiosira hyalina* (Grunow) Gran |  |
| *Thalassiosira hyperborea* (Grunow) Hasle |  |
| *Thalassiosira nordenskioldii* Cleve |  |
| *Thalassiosira* spp. |  |
